# Proceedings of the Fourth Annual Deep Brain Stimulation Think Tank: A Review of Emerging Issues and Technologies

**DOI:** 10.3389/fnint.2016.00038

**Published:** 2016-11-22

**Authors:** Wissam Deeb, James J. Giordano, Peter J. Rossi, Alon Y. Mogilner, Aysegul Gunduz, Jack W. Judy, Bryan T. Klassen, Christopher R. Butson, Craig Van Horne, Damiaan Deny, Darin D. Dougherty, David Rowell, Greg A. Gerhardt, Gwenn S. Smith, Francisco A. Ponce, Harrison C. Walker, Helen M. Bronte-Stewart, Helen S. Mayberg, Howard J. Chizeck, Jean-Philippe Langevin, Jens Volkmann, Jill L. Ostrem, Jonathan B. Shute, Joohi Jimenez-Shahed, Kelly D. Foote, Aparna Wagle Shukla, Marvin A. Rossi, Michael Oh, Michael Pourfar, Paul B. Rosenberg, Peter A. Silburn, Coralie de Hemptine, Philip A. Starr, Timothy Denison, Umer Akbar, Warren M. Grill, Michael S. Okun

**Affiliations:** ^1^Department of Neurology, Center for Movement Disorders and Neurorestoration, University of FloridaGainesville, FL, USA; ^2^Department of Neurology, and Neuroethics Studies Program, Pellegrino Center for Clinical Bioethics, Georgetown University Medical CenterWashington, DC, USA; ^3^Department of Neurosurgery, Center for Neuromodulation, New York University Langone Medical CenterNew York, NY, USA; ^4^J. Crayton Pruitt Family Department of Biomedical Engineering, University of FloridaGainesville, FL, USA; ^5^Department of Neurology, Mayo ClinicRochester, MN, USA; ^6^Department of Bioengineering, Scientific Computing and Imaging Institute, University of UtahSalt Lake City, UT, USA; ^7^Department of Neurosurgery, University of Kentucky Chandler Medical CenterLexington, KY, USA; ^8^Department of Psychiatry, Academic Medical Center, University of AmsterdamAmsterdam, Netherlands; ^9^Department of Psychiatry, Massachusetts General HospitalBoston, MA, USA; ^10^Asia Pacific Centre for Neuromodulation, Queensland Brain Institute, The University of QueenslandBrisbane, QLD, Australia; ^11^Department of Anatomy and Neurobiology, University of Kentucky Chandler Medical CenterLexington, KY, USA; ^12^Departments of Psychiatry and Behavioral Sciences and Radiology and Radiological Sciences, Johns Hopkins University School of MedicineBaltimore, MD, USA; ^13^Division of Neurological Surgery, Barrow Neurological Institute, St. Joseph's Hospital and Medical Center PhoenixArizona, AZ, USA; ^14^Department of Neurology and Department of Biomedical Engineering, University of Alabama at BirminghamBirmingham, AL, USA; ^15^Departments of Neurology and Neurological Sciences and Neurosurgery, Stanford UniversityStanford, CA, USA; ^16^Department of Psychiatry, Emory University School of MedicineAtlanta, GA, USA; ^17^Electrical Engineering Department, University of WashingtonSeattle, WA, USA; ^18^NSF Engineering Research Center for Sensorimotor Neural EngineeringSeattle, WA, USA; ^19^Department of Neurosurgery, VA Greater Los Angeles Healthcare SystemLos Angeles, CA, USA; ^20^Department of Neurology, University Clinic of WürzburgWürzburg, Germany; ^21^Department of Neurology, University of California San FranciscoSan Francisco, CA, USA; ^22^Department of Neurology, Baylor College of MedicineHouston, TX, USA; ^23^Department of Neurological Sciences, University of FloridaGainesville, FL, USA; ^24^Departments of Neurological Sciences, Diagnostic Radiology, and Nuclear Medicine, Rush University Medical CenterChicago, IL, USA; ^25^Division of Functional Neurosurgery, Department of Neurosurgery, Allegheny General HospitalPittsburgh, PA, USA; ^26^Department of Neurology, New York University Langone Medical CenterNew York, NY, USA; ^27^Psychiatry and Behavioral Sciences, Johns Hopkins Bayview Medical Center, Johns Hopkins School of MedicineBaltimore, MD, USA; ^28^Graduate Program in Neuroscience, Department of Neurological Surgery, Kavli Institute for Fundamental Neuroscience, University of California, San FranciscoSan Francisco, CA, USA; ^29^Medtronic ModulationMinneapolis, MN, USA; ^30^Movement Disorders Program, Department of Neurology, Alpert Medical School, Rhode Island Hospital, Brown UniversityProvidence, RI, USA; ^31^Department of Biomedical Engineering, Duke UniversityDurham, NC, USA

**Keywords:** deep brain stimulation, Parkinson's disease, Alzheimer's disease, closed-loop, depression, post-traumatic stress disorder, Tourette syndrome, DARPA

## Abstract

This paper provides an overview of current progress in the technological advances and the use of deep brain stimulation (DBS) to treat neurological and neuropsychiatric disorders, as presented by participants of the Fourth Annual DBS Think Tank, which was convened in March 2016 in conjunction with the Center for Movement Disorders and Neurorestoration at the University of Florida, Gainesveille FL, USA. The Think Tank discussions first focused on policy and advocacy in DBS research and clinical practice, formation of registries, and issues involving the use of DBS in the treatment of Tourette Syndrome. Next, advances in the use of neuroimaging and electrochemical markers to enhance DBS specificity were addressed. Updates on ongoing use and developments of DBS for the treatment of Parkinson's disease, essential tremor, Alzheimer's disease, depression, post-traumatic stress disorder, obesity, addiction were presented, and progress toward innovation(s) in closed-loop applications were discussed. Each section of these proceedings provides updates and highlights of new information as presented at this year's international Think Tank, with a view toward current and near future advancement of the field.

## Introduction

The Fourth Annual Deep Brain Stimulation (DBS) Think Tank convened in Gainesville, FL, on March 9–11, 2016. In this summary we provide the meeting topics and expert updates, as well as important highlights in each area. DBS use has expanded in many neuropsychiatric areas and there is a need for an interdisciplinary approach incorporating neurologists, neurophysiologists, neuroscientists, neurosurgeons, psychiatrists, rehabilitation specialists, ethicists, members of industry, and engineers. The DBS Think Tank aims to be an annual forum that facilitates sharing, discussing, and debating the latest innovations and challenges in the field. This year's Think Tank focused on the regulatory process and advocacy; innovative techniques and indications; updates in the field of responsive DBS (closed-loop systems), as well as updates on associated advances in electrophysiology and sensor technology.

The overarching goal was not to produce an evidence-based summary or practice guidelines, but rather to engage participants toward addressing and solving unresolved issues that impede current and near-term research and translation of DBS. This approach has the potential to expand collaborative research, improve care and strengthen the field. The meeting, conducted in a think-tank style, afforded equal time to key speakers' presentations, and group roundtable discussions. The current proceedings of the Think Tank provide a summary and review of the developments, challenges, and opportunities in DBS research and its clinical translation.

## Development of an international registry and database of DBS for tourette syndrome

### Update

Tourette Syndrome (TS) is a complex neuropsychiatric disorder with multiple motor and vocal tics that can incur difficulty with social engagement and communications that can often be debilitating (Cheung et al., 2007; Kenney et al., 2008; Hanks et al., 2015). DBS has been explored in a subset of TS subjects with severely disabling symptoms. An international TS DBS registry and database was established in 2012 by investigators in the TS DBS field and the Tourette Association of America (TAA; previously the Tourette Syndrome Association, TSA; Deeb et al., 2016). The need for the registry and database was based on the relatively low number of cases of TS patients who have received DBS. The registry and database were therefore developed to facilitate pooling information on these cases to define and refine anatomical targets, develop management strategies, improve therapeutic outcomes, inform, and support regulatory agency approval, and ultimately, improve the quality of patient care.

Data are registered and securely stored at the University of Florida, which serves as the hub site. The registry and database enable collaborators to safely access and use the data for research and practice improvement. The project collects cases of TS who receive DBS from network sites, and encourages investigators to submit complete treatment and follow up data on every case. Data from multiple domains, including demographic information, pre- and post-operative clinical measures, surgical measures, lead placement, DBS programming, and adverse events are registered.

**Figure 1:**
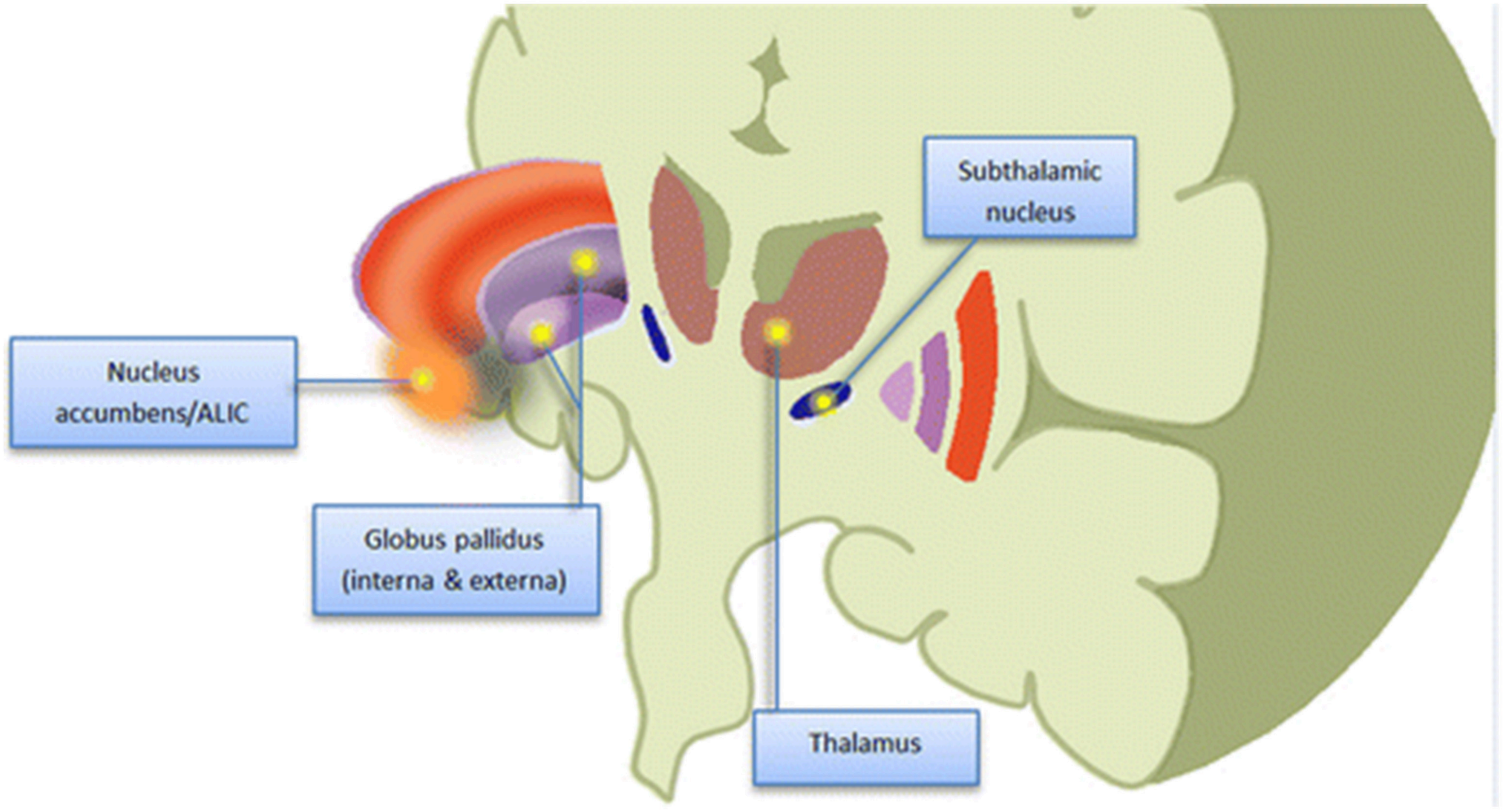
**Schematic (cartoon) representation of potential therapeutic targets of DBS for Tourette Syndrome**. Figure is not drawn to scale. ALIC, anterior limb internal capsule (From Malaty and Akbar, 2014; with permission).

Multiple brain sites have been targeted for DBS in TS (Figure 1; Malaty and Akbar, 2014).

By March, 2016 there have been 149 cases from 16 different institutions registered. There were 94 cases targeting thalamic regions (centromedian, parafascicular nuclei); 23 cases with anteromedial pallidal targets; 41 cases with posteroventral pallidal targets; and 2 cases with nucleus accumbens/ventral capsular/ventral striatum targets. Interestingly, the age at the time of surgery has been decreasing for TS DBS. This has been reflected in development of revised guidelines, which now no longer advocate that TS patients be a minimum age of 25 in order to be considered as viable candidates for DBS. Indeed, TS patients younger than 18 years of age have had good clinical outcomes following DBS treatment (Schrock et al., 2015). However, data also reveal that multiple co-morbidities, including obsessive-compulsive disorder (OCD), major depressive disorder and attention deficit disorder (ADD), exist in the TS population, and it is intended that the database and registry will provide further information to elucidate how these conditions affect, and are affected by DBS intervention.

To date, one of the most significant barriers to accruing a relatively complete evidence base has been difficulties in acquiring longitudinal datasets. Many records are missing information regarding co-morbid conditions and motoric and phonic tic follow-up scores at 6, 12, and 24 months. Additionally, sub-score collection has been incomplete for tic scales (Yale Global Tic Severity Scale YGTSS), and more data are required on the actual DBS settings and their changes over longitudinal follow up.

Developing a more finely grained understanding of the problems with the technology, physiological effects, and adverse events will be critically important to map the future of DBS therapy, and new forms for effect and event recording matching the Food and Drug Administration (FDA) standards have been implemented. Adverse event reporting has included surgical, psychiatric, cognitive and general events. Preliminary data have revealed a higher than anticipated number of device explantations and issues precipitating device removal and these need to be further explored. Servello et al. (2016, 2011) have shown TS DBS to be associated with increased infections and hardware issues, but in some cases, the devices were removed due to resolution of symptoms. In a limited number of cases, post-operative lead location measurements have been made available, and increasing such data will be important to the registry. Multiple approaches have been suggested and implemented to improve the collection of data across the numerous centers and groups that provide DBS treatment for TS. For example, quarterly reminder messages will now be sent to contributors in order to acquire heretofore-missing data fields, and a dashboard has been developed to allow secure, multi-site access to data.

The registry and database effort has been initially successful in collecting information on safety of DBS in the TS population, understanding preliminary effectiveness, and in driving better outcomes. A planned objective is to explore if and how the database could—and should—be utilized to inform and support more a more facile method for obtaining of humanitarian device exemptions (HDE), or other approval for DBS use from other international regulatory agencies. Here a number of key questions were posed that were regarded as important to leveraging DBS in other potential areas of clinical application, as well. These questions included: What obtaining HDE approval would mean to scope and extent of research in the field. What lessons can be learned from the OCD HDE experience? If HDE approval does not prove to be a viable next step, how might the registry and database be employed to help refine large randomized clinical trials? What types of metrics [e.g., predisposing features; clinically response measures; quality of life (QoL) indicators] will be important to characterize a good responder? Is there a role for subjective narrative input from each participating subject?

**Highlights**

- The TS DBS registry and database effort started in 2012 to bridge the knowledge gap in the use of DBS in TS subjects.
- More consistent and extensive data collection is needed to improve clinical outcome assessments, lead locations, programming parameters and adverse event reporting.
- Future areas of effort include:
  ◦ Studying the viability and impact of obtaining HDE approval in TS DBS and its implications.
  ◦ Characterizing TS subject phenotypes and meaningful clinical metrics.
  ◦ Comparing outcomes of different surgical targets and stimulation paradigms.

### Registering lead locations and how to use the data

The registry and database can serve as an expansive resource of diverse types and levels of information that will be essential to further define and refine the possible use(s) of DBS. For example, there is an important role for data from functional magnetic and diffusion tensor and kurtosis-imaging studies to further systematically depict lead location(s), and changes in the activity of anatomical nodes and tracts that may be involved in, and/or subserve observed clinical outcomes and effects.

There are several laboratory-based tools for predicting and reconstructing DBS effects. However, these tools are often difficult to use and incur a relatively steep user-learning curve. Developing simple systems to disseminate three-dimensional (3-D) interactive models could provide means toward more useful and user-friendly toolkits. One proposed approach toward this objective is to incorporate plotting and predictive functions into an interactive 3-D model. The method would employ a visualization component that provides volume rendering as well as surface renderings. The results would reveal the effect-size on specific clinical outcomes (such as bradykinesia) and would represent results as a function of stimulation-location. The informatics component allows the user to use a widget to query a position in space that will reveal different visualizations of the outcome data associated with stimulating a specific point in space. Clinical effect sizes for various effects can also be extracted from the data obtained.

A composite figure of actual lead locations in the subthalamic nucleus was produced using data from a multi-center DBS clinical trial. It revealed significant variability in lead location and trajectory across the different centers, despite targeting the same structure, and region (subthalamic nucleus). Analyzing the variability in lead location will be critical, as it will allow more accurate site specific correlation of lead placement and clinically relevant (objective and subjective) effects of DBS in treating different signs and symptoms of various disorders.

Future steps in developing imaging databases include measures for insuring patient (and clinician) anonymity, consideration of a data-use embargo period, and defining the terms of use of information in the database. Participants in the Think Tank proposed the possibility of a central data repository of images and lead locations, to which practitioners could upload individual scans to be used for comparisons andbenchmarks.

To be sure, the collection and assimilation of various types and extent of data represent challenging tasks, and opportunities. At present, a number of computational tools are available to facilitate data collection and sharing. One such tool, developed by the Center for High Performance Computing at the University of Utah, enables use of a protected data environment platform to allow collection of sensitive, personal health information. This organized, scalable infrastructure can be used to host RedCap® and imaging software that enable differing types of data from providers, patients, and caregivers to be entered and analyzed. Ongoing efforts will be focused upon developing this and other big data platforms to optimize collection, integration, use, and modeling of diverse information.

**Highlights**

- Any database effort needs to establish the short-term, medium term, and long-term goals.
- Lead location in the TS DBS database effort is a bedrock in understanding outcomes.
- Steps are needed to improve collaboration and eliminate obstacles.
  ◦ Tools are available to facilitate sharing of interactive and predictive 3-D models.
  ◦ The think tank participants recommended the development of a central data repository of lead location images.

### Quantifying economic impacts of deep brain stimulation

Since 1999, a small number of patients worldwide have received DBS for severe TS (Ackermans et al., 2008). Although, clinical results have been promising, establishing clinical effectiveness is not always sufficient to ensure investment in new medical technology.

The Center for Movement Disorders and Neurorestoration at the University of Florida maintains an international database of patients with severe TS who have received DBS (n ≈ 150). While clinical data is collected pre- and post-DBS, to date economic data have not been collected. When medical treatments must compete aggressively for a limited pool of healthcare resources, well-designed economic evaluation is essential to ensure that necessary resources are directed toward treatments that offer the best outcomes. In light of this, a comprehensive economic evaluation of DBS for TS is planned.

A survey of patients and treating medical practitioners will be undertaken to collect data necessary for economic evaluation. Patients will be surveyed for indirect medical costs, including workforce participation and health related quality of life (HRQoL) using a validated instrument (e.g., SF-36 or EQ-5D). The treating medical team will be asked to report direct medical costs and relevant post-operative clinical data (e.g., verification of the neuroanatomical location of the DBS electrodes, etc.). Direct medical costs will include the costs of DBS hardware, surgery, inpatient stay, neurostimulator titration, and post-operative complications. Quality-adjusted life years (QALYs), a generic metric of HRQoL, is routinely used as a summary measure of health outcomes in cost-utility analyses (CUA) (Drummond et al., 2015). A QALY of one denotes a year of life lived in perfect health. Years lived in less than perfect health are scored less than one. Health policy analysts deem cost per QALY ratios, less than some designated threshold, as being cost effective. Thresholds between nations will vary, and can be approximated by the gross domestic product (GDP) per capita (Marseille et al., 2015). For example, ratios of US $50,000 per QALY (Grosse, 2008) and £20,000–£30,000 per QALY (McCabe et al., 2008), are used in the United States and United Kingdom, respectively. Post-operative QALYs will be derived from reported HRQoL sub-item scores. Pre-operative QALYs will be hindcast, using coefficients obtained from statistical analysis, which regress clinical variables on post-operative QALYs (Dodel et al., 2010; Müller-Vahl et al., 2010). Costs and QALYs will then be analyzed and incremental cost effectiveness ratios (ICERs) reported. Other methods of analysis, such as the Minimal Clinically Important Difference (MCID) may be valuable. We believe that such findings will represent an important first step to elucidating health outcomes' afforded by DBS, and to informing appropriate investment in DBS technologies and practices.

**Highlights**

- It is planned that economic data should be collected to establish the cost-effectiveness of DBS as a treatment for severe TS.
- Technical (e.g., post-operative electrode placement), as well as direct and indirect health costs plus a generic measure of HRQoL data should be collected.

### Regulatory processes and translational viability: time for a change?

#### Investigational use of DBS in clinical practice

The overarching goal of the US Food and Drug Administration (FDA) regulation process is to establish that any and all drugs and devices provided for medical care are safe and technically sound. In the United States, device trials utilizing either a non-approved or an approved device to be used for a non-approved indication require an investigational device exemption (IDE) to be granted from the FDA. Failure to obtain an IDE will preclude most Institutional Review Boards (IRBs) from approving prospective studies of off-label use of devices. Both the IDE and HDE entail considerable detail in scope, application, review and guidance, and such stringency is necessary and important to determine and to assure probity in applications of technology. Moreover, whereas IDEs can be obtained (by industry) for industry-sponsored device trials, investigators are required to obtain the IDE in non-industry sponsored trials; this can be- and frequently is—an arduous, and time- and cost-expensive process.

In recent years, IDE and HDE applications, review and approval have become considerably more facile and efficient; this is a notable improvement—and a step in the right direction. However, as regards to DBS, it may be that aspects of the overall structure and certain specifics of the IDE and HDE are not well suited to meet the contingencies (and exigencies) of actual clinical use, particularly in light of interest in exploring if and how DBS may be of clinical benefit in the treatment of an expanding number of neuropsychiatric conditions (as detailed elsewhere in this report). For example, the current regulatory framework necessitates filing and securing an IDE as a first step in investigator-initiated research (IIR) and/or other off-label use of DBS in those cases where other approaches have been shown to be ineffective or untenable, and for which DBS may prove to be viable as “humanitarian care.” In such instances, it may be that the proverbial cart precedes the horse, and the HDE might be more practical and valuable given both the nature of the disorder and treatment, and the value of the HDE in establishing a basis for further (and/or expanded) application, as supportable by an IDE.

Moreover, while both IDE and HDE establish parameters for using DBS in practice, neither regulatory mechanism establishes or enforces a basis for provision of economic support necessary for right and good use-in-practice. As recent work has demonstrated, non-payment of insurance costs for pre-certified DBS interventions has been, and remains a problem of considerable concern (Rossi et al., 2016a,b). Absent resources to provide: (1) DBS as a demonstrably-important or necessary treatment option for those subjects with conditions that are non-responsive to, or not candidate for other therapeutic options, and (2) continuity of clinical services as required, the sustainability of this neurotechnology may become questionable (Rossi et al., 2014). We see this as contrary and counter-productive to recent federal incentives to maximize benefits of translating extant and new neurotechnologies into clinically-relevant and affordable care and to implementing precision medicine.

In the main, we applaud actions taken by the FDA to date that have streamlined the IDE and HDE process. Yet, while certain aspects of the IDE and HDE mechanisms may be in order, apt, and valuable for regulating use of DBS, others may require re-examination, revision or replacement, so as to remain apace with developments in the field, and needs and necessities (of both subjects and clinicians) in practice. In this vein, we recommend further study of: (1) the scope and tenor of the IDE and HDE mechanisms to determine their independent and interactive benefit (as noted above); (2) whether and which aspects of the current IDE/HDE process are effective and efficient, and which are not; (3) what aspects need to be retained and fortified, revised or replaced; (4) what is entailed in these revisions/replacements; and (5) if and how regulatory, policy and legal processes can and should be aligned with, directive toward, and supportive of and by concomitant changes in standard of care guidelines and federal insurance structure (Fins et al., 2012). A number of possible alterations to the IDE process were addressed, which may streamline application and granting of regulatory approval. These include: removal of the right of reference letter (ROR) requirement; improved alignment of federal grant mechanisms and regulatory process, and institution of mechanisms for Centers for Medicare and Medicaid Services (CMS) and private insurance payment to support costs incurred by patients involved in these trials. Proposed alternatives to IDE-sponsored trials were also addressed, including the viability and value of retrospective analyses of multiple case series, and large scale, multi-site single case analyses, which could be facilitated through the use of currently available computational tools (e.g., the *AvesTerra* System; see: http://osvpr.georgetown.edu). that enable massive data assimilation and integration, both in concert with, and independently of a registry mechanism. Important to this effort would be the development of both a governmental-commercial enterprise to guide industrial efforts in neurotechnology (e.g., a National Neurotechnology Initiative; NNTI), as well as the establishment and enactment of federal laws (e.g., a neurological information non-discrimination act; NINA) to govern potential use(s) of information obtained through DBS and related neurotechnologies together with extant and novel big data initiatives (Kostiuk, 2012; DiEuliis and Giordano, 2016). We believe that while establishing this “translational estate” will require significant effort; it represents a worthwhile endeavor toward the achievement of genuine and durable progress in the development and use of neurotechnology in clinical practice.

## DBS innovations

### Tourette syndrome

As noted, much of the more innovative work to date has (and remains) focused upon studying the viability and value of DBS for the treatment of Tourette syndrome. While the exact causes of TS remain unknown, recent neuropathology neuroanatomical investigations have collectively implicated dysfunction of corticostriatal and thalamocortical circuits thought to play a role in the generation of abnormal motor programs, possibly due to aberrant thalamic disinhibition (Albin and Mink, 2006). The collection of neural activity from the awake and behaving human TS patients will offer new and vital insights to the underlying neurophysiology of tic generation. To this end, next generation DBS devices, such as the *Neuropace RNS* and Medtronic *Activa PC*+*S* enable recording of electrophysiological signals from both the implanted depth electrodes, as well as acutely placed electrocorticography (ECoG) strips.

**Figure 2:**
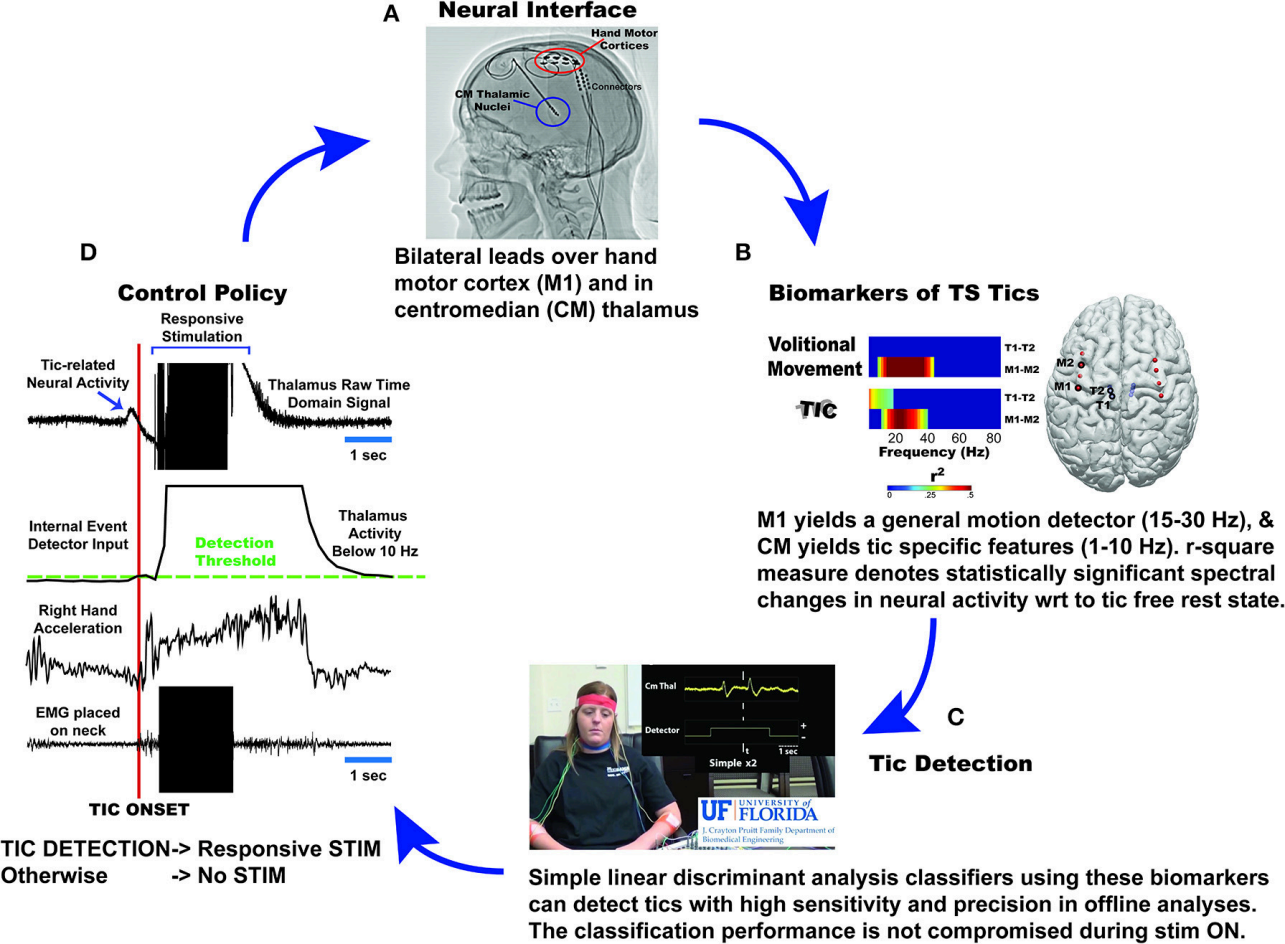
**Diagrammatic depiction of the University of Florida approach to implementing chronic responsive DBS therapy for Tourette Syndrome**. Current experience with two patients with TS, who received bilateral centromedian (CM) thalamus depth leads and bilateral subdural grid implantation over their hand motor cortex **(A)**, led to the discovery of tic specific features in CM thalamus (1–10 Hz) and motion detection features in hand motor cortex (15–30 Hz; beta rhythm) **(B)**. A combination of these two features yielded highest detection of tics and differentiation from voluntary movements in linear discriminant analysis classifiers **(C)**. These classifiers are embedded in PC+S and send control signals to Nexus- E stimulation engine **(D)**. Once the detectors sense presence of tic related activity, stimulation will be activated to deliver stimulation to optimize therapeutic effects/outcomes.

An unpublished study was presented that examined the effects of DBS on two patients with severe, medication refractory TS. Patients were implanted with bilateral Medtronic *Activa PC*+*S* devices. Depth leads were placed in the centromedian-parafascicular nucleus of the thalamus (CM) and ECoG strips were placed over the precentral gyrus to cover the hand primary motor cortex (M1). Experiments consisted of separate interleaved trials in which patients were instructed to: (1) tic freely, (2) suppress tics (baseline), and (3) execute volitional movements (e.g., shaking hands rapidly, opening and closing hands, raising arms up, and down, talking). Post-operatively recorded data suggested that M1 yields a general motion detector (15–30 Hz), whereas CM yields tic-specific features (1–10 Hz). A human tic detector, based on support vector machines was constructed during each post-operative visit (for a period of 6 months). Three types of tics were recorded including simple, complex, and long complex tics. Long complex tics were shown to be concurrent with a consistently detectable thalamocortical signature. Short complex tics were more difficult to detect than long complex tics, and simple tics were the most difficult to detect. Acute trials of closed loop stimulation using the Medtronic Nexus-E platform are currently underway. The proposed system is presented in Figure 2.

**Highlights**

- The initial RNS study in TS patients revealed that good benefit in tic control can be achieved with scheduled stimulation as compared to continuous stimulation.
- LFP-ECoG neurophysiological testing identified a correlation between tic activity and appearance of a 10 Hz narrow band signal.
- Targeted stimulation using the 10 Hz band as signal resulted in tic improvement (preliminary results).

### Summary of use of DBS to treat epilepsy

Epilepsy, the result of the hyper-synchronization of firing of neurons, creates “fragile” neurological networks that tend to cycle. Multiple modalities of neurostimulation have been developed to modulate burst and cycling activity in epileptic patients (Krishna et al., 2016). In addition to DBS, techniques such as vagus nerve stimulation (VNS), which engages afferents of peripheral nervous system input to activate vagal pathways, can alter firing patterns of brain networks involved in ictal discharges and cycling. As well, the use of other neuromodulatory techniques, such as responsive neurostimulation (RNS), has been explored (Chang et al., 2015).

These approaches differ in their modulatory effects upon cycling time, burst duration, frequency, and amplitude. In addition, the locations of the VNS and DBS electrode placement (at the anterior nucleus of the thalamus) are the same in all patients while RNS employs a variety of possible placement sites. The site of RNS is dependent upon identifying the epileptogenic locus of nodes involved in a specific patient. In most cases, this has been shown to be cortical gray matter. However, patients with long-standing refractory epilepsy have been shown to develop areas of secondary epileptogenesis, possibly through kindling. To better manage multiple epileptogenic loci in this population, stimulation of the affected circuitry (white matter) rather than the epileptogenic gray matter is being considered (Girgis and Miller, 2016). In these studies, it has been shown that microelectrode recording and modification of the area of stimulation can achieve differential, acute and chronic effects on the involved neurocircuitry. Chronic effects appear to be related to stimulation-induced plasticity, and may engage trophic mechanisms in that they subserve (at least some component of) the therapeutic outcomes of neurostimulation in this patient population.

**Highlights**

- Studies of the mechanisms and effects of DBS in treating epilepsy can be useful to both an expanded understanding of DBS and brain pathology, and can synergize the development of other types of neuromodulation.
- Studies of DBS (and VNS and RNS) reveal the importance of determining and identifying anatomical targets (gray matter) vs. circuit targets (white matter).
- Brain stimulation can exert acute and chronic effects, the latter being related to neuro-plasticity and trophic effects.
- The role of multi-site, multi-electrode pre- and intra-operative recording is essential to advancing understanding and improvement of neuromodulation approaches to the treatment of epilepsy; however, how findings from studies of the use of DBS, VNS, and RNS may translate to broader applications of these techniques remains a subject of continuing speculation.

### Novel DBS settings—biphasic pulses and beyond

DBS signal delivery is a rapidly progressing field. Recent innovations in DBS signal delivery (Fasano and Lozano, 2015) include regulated current vs. regulated voltage waveforms (Lempka et al., 2010; Preda et al., 2016), differing stimulation waveforms (Foutz and McIntyre, 2010; Wongsarnpigoon and Grill, 2010), and different temporal patterns of stimulation (Brocker et al., 2013; Adamchic et al., 2014).

Studies have repeatedly demonstrated the enhancement of the therapeutic window with lower DBS pulse widths (Moro et al., 2002; Volkmann et al., 2014). High frequency stimulation (HFS; >100 Hz) has generally been considered to be effective for mitigating certain signs and symptoms of Parkinson's disease (PD), but low frequency stimulation (LFS; <100 Hz) has yielded contradicting results. LFS <50 Hz has been shown to be harmful resulting in worsening bradykinesia and tremor (Moro et al., 2002). Stimulating at individualized gamma frequencies (30–90 Hz) improved PD symptoms, with outcomes that were similar to those produced by HFS (Tsang et al., 2012). These findings suggest that LFS can be effective provided that it is appropriately matched to subject's individualized gamma frequency patterns associated with movement. Irregular patterns of stimulation have also been studied in computational models, non-human primates, and human patients. While there are some irregular patterns that seem to be as effective as—or more effective than—regular HFS, evidence for human testing remains limited. A recent randomized, blinded pilot study of nonconventional DBS patterns and pulses—the first reported study of its kind—tested 3 essential tremor and 8 PD clinically-optimized patients in a clinic setting (Akbar et al., 2016). Of the settings tested, the nonconventional biphasic pulse (equal and opposite, active recharge phase) was shown to be more effective than the clinically optimized settings. Of course, it may be premature to draw firm conclusions about the effectiveness of this pulse shape based upon the results of this small pilot cohort, but such findings are both of great interest and promising in their implications for the viability and effectiveness of novel pulse and pattern parameters. Additional studies to further investigate these possibilities and to address the potentially short washout interval are underway.

**Highlights**

- A number of techniques of stimulation are available including differing stimulation waveforms and current.
- There is a differential therapeutic effect of the various stimulation parameters in DBS that appears to be related to the underlying disease process (e.g., PD, dystonia). A recently published pilot trial to assess different stimulation parameters in PD and essential tremor subjects revealed significant improvement induced by symmetric biphasic pulse stimulation.
- Ongoing unresolved issues include the effect of differing pulse and pattern settings on battery drain, requisite washout time, and biophysical changes induced in affected neural nodes and circuits.

### Development of DBS sensors

DBS surgery provides an investigational opportunity for use of electrophysiological and/or neurochemical recording techniques. Such approaches can: (1) aid in DBS lead placement, (2) provide additional information about disease states, and (3) potentially enable future development of techniques to better control and fine-tune DBS therapies including closed-loop control (Herrington et al., 2016). In addition, DBS surgery provides a vector to introduce stem cells, autologous transplants, and/or gene modification. We have termed the conjoined use of these approaches *DBS Plus*.

Future directions in DBS have been proposed to incorporate real-time monitoring of field potentials/unit activity, and *in vivo* assessment of neurotransmitter release and turnover (Paek et al., 2013). Such combinatory approaches could be used to further define brain networks affected in disease processes, which could serve to elucidate target sites for current and future applications of DBS (including closed-loop systems) to more effectively—and automatically—program, control, and modify stimulation parameters (Grahn et al., 2014).

These iterations are currently under development. For example, RNS for epilepsy and a new DBS variant manufactured by Medtronic, the Brain Radio, implement simultaneous field potential recordings that are coupled to neural stimulation. The use of simultaneous DBS and neurotransmitter measurement is being studied by Lee and coworkers in a Phase I investigation using fast-scan cyclic voltammetry recordings coupled to carbon fiber or boron doped diamond-like carbon microelectrodes (Bennet et al., 2016). Gerhardt, van Horne and colleagues are investigating (personal communication) the possible use of glucose and glutamate as chemical biomarkers for control of DBS. Current preclinical studies support that oxygen and glutamate measures can be used to reveal both tonic and phasic changes in neuronal systems that may be indicative of trait- or state-dependent properties (Stephens et al., 2014). A persistent technical impediment to these types of studies is the difficulty of long term monitoring of neurochemistry *in vivo*. As well, it remains unclear if and how *in vivo* neurochemical monitoring can be durably yoked to DBS. The combined use of electrophysiological recordings and real-time neurochemical monitoring show considerable value for closed-loop control of RNS technology for epilepsy, and for closed-loop control of DBS therapy for PD. Nevertheless, given the early stage of these developments, it will be important to continue studies of real-time neurochemical monitoring for use in both open- and closed loop DBS applications pursuant to advancing these approaches toward more broadly applied clinical translation.

**Highlights**

- We introduce the concept of *DBS Plus* to describe the incorporation of additional treatment and recording modalities (e.g., stem cells, gene modification, neurochemical monitoring, etc.) during DBS surgery.
- Multi-modality monitoring can be important to identifying neural circuity involved in various pathologies and DBS effects, and in these ways can facilitate more accurate electrode target placement.
- These *DBS Plus* approaches show promise in the further development of closed-loop systems.

### DBS for Alzheimer's disease

Alzheimer's disease (AD) is the most common cause of dementia worldwide (Scheltens et al., 2016). Current focus of treatment for AD treatment has pharmacotherapy aimed at modifying acetylcholinesterase activity, N-methyl D aspartate receptor activation, and more recently, production or deposition of beta-amyloid or tau proteins. The limited evidence for symptomatic benefit or slowing of disease progression from these approved and investigational treatments, as well as the side effects reported, support pursuing other avenues of intervention (Winblad et al., 2016).

The importance of developing approaches to modulate cortical and hippocampal circuits affected in AD was the impetus for a phase I study of DBS targeting the fornix (Laxton et al., 2010). The choice of the fornix as the target was based upon serendipitous observation of improved spatial and verbal learning and memory functions in patients who received DBS leads in the hypothalamus for obesity management. In the phase I study, continuous fornical stimulation produced sustained increases in cortical metabolism at 1 month and 1 year post-operatively. Further, increased functional connectivity was observed in two orthogonal networks: a frontal-temporal-parietal-striatal-thalamic network and a frontal-temporal-parietal-occipital-hippocampal network. These increases in functional connectivity were greater than effects produced by 1 year of pharmacotherapy (with cholinesterase inhibitors) and were in contrast to metabolic reductions and decreased functional connectivity seen in the 1 year course of AD. Higher cortical metabolism prior to initiation of DBS, as well as increased metabolism after 1 year of DBS, were correlated to better outcomes in global cognition, memory, and quality of life indices (Smith et al., 2012). A multi-center, double-blind, randomized, and controlled Phase II trial of 42 mild probable AD patients—the *ADvance trial*—was conducted (Lozano et al., 2016). In this study, the mean age of subjects was 68.2 ± 7.8 years (younger than the AD population, but similar to the age range of AD patients enrolled in clinical trials; Leinonen et al., 2015). Average disease duration since diagnosis was 2.3 ± 1.7 years. Electrodes were implanted in all patients, but half of the patients did not receive stimulation for the first 12 months, and were subsequently crossed over to active stimulation. The trajectory was trans-ventricular and implantations of Medtronic hardware were bilateral (Ponce et al., 2016). Stimulation was applied using extant PD protocols, with a frequency of 130 Hz, pulse width of 60 μs, and voltage set at 50% of that at which side effects (e.g., autonomic or cognitive changes) were seen, with a maximum test voltage set at 7 V and maximum continuous voltage of 3.5 V. There was no noted acute decline in cognition after 1 month of surgery. Consistent with results of the prior Phase I study, persistent increases in metabolism were observed in the group receiving stimulation (i.e.,- the ON group) after 6 and 12 months of DBS of the fornix, in contrast to the OFF group that showed decreased metabolism (7–13%) across all regions assessed. The primary goal was safety; the safety profile of the procedure was acceptable and comparable to pharmacologic therapies. There were some short-term side effects related to the surgery, as well as some psychiatric side effects (as expected following DBS). None of the subjects had persistent side effects or complications at 12 months follow-up.

Secondary goals were to evaluate the preliminary efficacy of therapy. These secondary end points were not met, although *post-hoc* analysis of subgroup evaluations based upon age showed that when patients 65 years of age or older were analyzed separately, greater increases in metabolism were observed in the ON group compared to those under age 65 (14–20% across regions over age 65). The subgroup aged <65 years had worsening clinical scores, while the subgroup aged >65 years showed improvement in clinical scores. The clinical scores used for this analysis included the Clinical Dementia Rating (CDR) and Alzheimer's Disease Assessment Scale—cognitive subscale (ADAS-Cog). In this older cohort, increased metabolism with fornical DBS was observed in the temporal and parietal cortices and hippocampal regions affected by AD, as well as in sensory and motor cortical regions that are relatively spared in this disorder. Functional connectivity and correlational analyses are currently in progress to determine the networks affected by DBS that are involved with clinical improvement, and the relationship between the metabolic and structural brain alterations associated with DBS to the fornix.

Other potential targets for DBS treatment of AD have been assessed and include the nucleus basalis of Meynert and the entorhinal cortex. Further studies are needed to: (1) clarify stimulation parameters for various brain regions and networks that can be targeted to mitigate signs and symptoms of AD; (2) define mechanisms through which DBS produces therapeutic and side effects in AD patients, and (3) to enable more accurate subject identification and selection.

**Highlights**

- DBS of the fornix has been shown in phase 1 studies to be associated with metabolic and clinical changes.
- The *ADvance* trial, a recently completed phase 2 study, assessed the safety of fornix DBS in AD, that demonstrated:
  ◦ No significant long-term complications,
  ◦ No acute cognitive decline after DBS surgery,
  ◦ Age-dependent effects with patients over age 65 achieving better outcomes,
  ◦ Concerns about bilateral simultaneous implantation,
  ◦ The need to further identify “optimal” stimulation parameters,
  ◦ Need for further study before considering fornical DBS as a viable treatment for AD in clinical practice.

## Closed-loop DBS

### Introduction

Existing DBS devices continuously stimulate their target structures regardless of the actual level of pathological activity. This can result in stimulation induced adverse effects, habituation, short battery life, and the need for labor-intensive programming sessions by a neurologist. Closed loop DBS enables simultaneous feedback and feedforward control of stimulation parameters that can afford a high level of precision and individual modification to variations in brain state. A major consideration of closed loop DBS is determination of the input signal. Recording brain signals in different therapeutic conditions (on and off DBS; on and on medication) has led to a better understanding of pathophysiology underlying PD, TS (discussed above) and major depression. This work results in the identification of disease markers that might be used as control signals for closed-loop DBS algorithms.

### Parkinson's disease

Published work has focused on the use of a beta-band signal (13–30 Hz) as a control signal (Little et al., 2013). However, the beta band is somewhat limited as a control signal by the influence of normal movement upon the signal fidelity. In light of this, current work is aimed at identifying oscillations that are outside of the beta-band that may be useful as markers. One, a narrow band gamma signal that has been defined as between 60 and 90 Hz, has been previously assessed as a surrogate signal using local field potential measurements (LFP) of the subthalamic nucleus (STN) (Brown et al., 2002; Cassidy et al., 2002). However, further study is required to more fully define the value of this, and other signals that can be utilized for optimized closed-loop control. Pursuant to such study, extensive neurophysiological work will be required. Work currently underway involves cortical recording with ECoG at the precentral gyrus/primary motor area and depth electrode recording at the level of the basal ganglia.

For example, to assess control signals in PD subjects with dyskinesia, researchers at the University of California at San Francisco (UCSF) group have implanted 5 patients with Medtronic Activa PC+S neurostimulators attached to a DBS lead (Medtronic 3389) in the STN and a 4-contact cortical ECoG strip (Medtronic Resume paddle) placed over the M1. Cortical and subcortical signals were collected over 2 years while patients were on and off therapeutic DBS as well as on and off dopaminergic therapy. During these recordings patients were resting with eyes open or engaged in a cued arm-reaching task (de Hemptinne et al., 2015). M1 and STN signals were recorded in a bipolar configuration at 800 Hz, stored and the PC+S downloaded non-invasively via telemetry and analyzed in the frequency domain.

**Figure 3:**
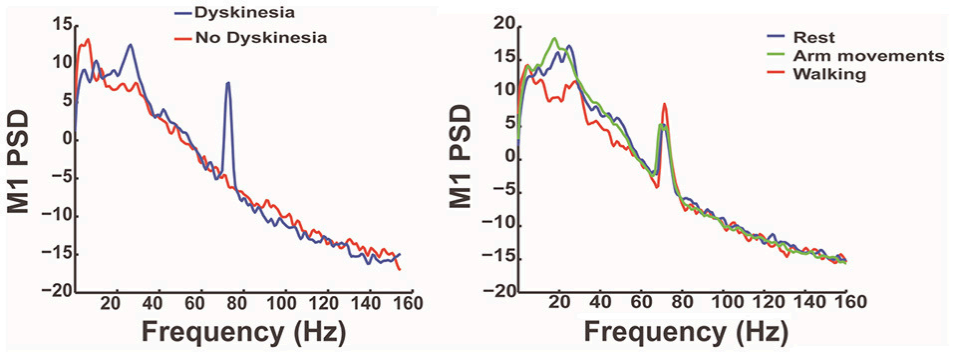
**The graphs depict the results of analysis of the M1 signal in the frequency domain in PD patients with dyskinesia**. It shows, in the graph on the left, that dyskinesia is associated with an increased neuronal synchronization in the gamma band (blue line) reflected as a narrow band peak. The graph on the right shows that this gamma-band signal is related to dyskinesia and independent of the functional state (rest, walking, or voluntary arm movement).

Using this unique data set (unpublished data), they found that periods of dyskinesia are associated with an increased neuronal synchronization in the gamma band (60–90 Hz; Figure 3). This excessive synchronization is reflected as a narrow band peak in the spectral power density of both M1 and STN signals, although less reliably detected in the latter. The emergence of this excessive synchronization occurs only in the presence of dyskinesia suggesting it as a marker of dyskinesia rather than a marker of the dopaminergic state. Interestingly, the cortical narrow-band gamma signal was shifted to half of the stimulation frequency when DBS was turned on, in the presence of dyskinesia only, which might be explained by a partial entrainment of axonal activity to stimulus pulses (Li et al., 2012). Contrary to broadband gamma activity, a non-oscillatory signal strongly affected by movement, the narrow-band gamma signal studied here was independent of the subject's “normal” voluntary non-dyskinetic movements. Other markers were studied including the coherence between the cortical and basal ganglia signal, as well as phase-coherence (unpublished data).

Narrow gamma band signals are part of a normal motif in brain connectivity allowing communication between multiple brain areas and alteration of these oscillations might results in dyskinesia as suggested by this study. Recordings in the motor cortex of a rodent model of PD identified a remarkably similar phenomenon in dyskinetic rats versus non-dyskinetic animals (Halje et al., 2012).

Given the predictable frequency at which this marker occurs, the simple method used to calculate it and the small impact of stimulation artifact of cortical signals, this biomarker is the ideal candidate to develop a closed-loop DBS algorithm. Therefore, the next step of this study is to develop closed-loop paradigms using this narrow-band gamma signal as a control signal and test it in PD patients with dyskinesia using the Medtronic Activa PC+S with the Nexus-D and E updates that allow for real time sensing and stimulation updates.

**Highlights**

- The use of beta-band subcortical oscillations in PD as a control signal is limited by the effects of voluntary movements and stimulation.
- Narrow gamma-band signal (60–90 Hz) appears to correlate with the dyskinetic state in PD subjects, is less affected by stimulation artifact and is independent of voluntary movements.
- Ongoing study to use the identified narrow gamma-band as a control signal for closed-loop DBS in PD patients for better control of dyskinesia.

### Depression

It has been ~10 years since the first proof-of-principle report supporting the efficacy of subcallosal cingulate (SCC) DBS to reduce signs and symptoms of treatment resistant depression (TRD; Mayberg et al., 2005). Initial selection of the SCC as a putative DBS target was principally based on converging findings from resting-state positron emission tomographic (PET) imaging studies of conventional antidepressant interventions, localization of depression-related circuits, and nodes using standard structural imaging methods, and trial-and-error behavioral testing of chronic stimulation at individual contacts on each implanted DBS electrode. As testing of DBS for treatment resistant depression has matured and expanded, neuroimaging continues to play a crucial role, with recent work now focused on refinement and optimization using multimodal methods combined with real-time behavioral and physiological metrics. These combinatory approaches afford more precise identification of optimal target locations in real time (Smart et al., 2015).

**Figure 4:**
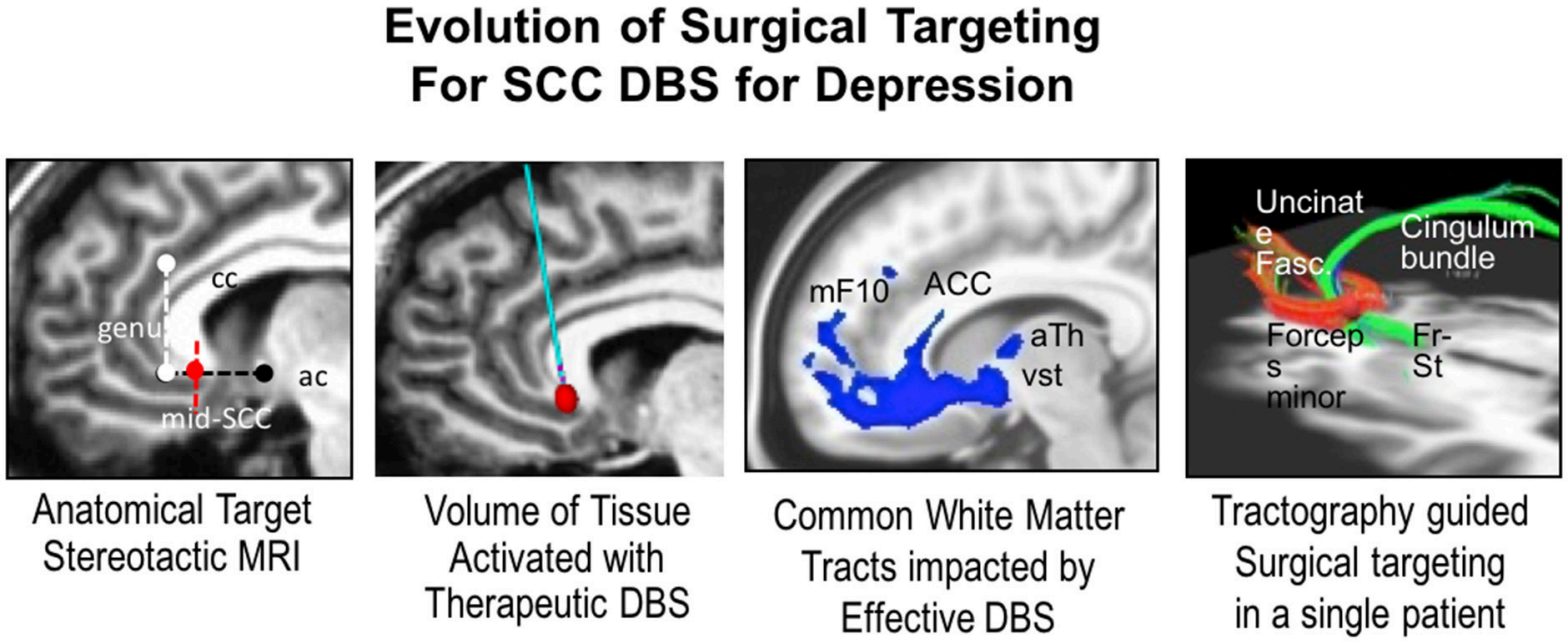
**The panes depict (from left to right) the evolution of surgical targeting of SCC in depression from, an anatomical “gray matter” target to identification of the “white matter” tracts activated, and finally tractographic data allowing identification of the involved pathways that elicit differing effects when targeted by DBS**. This approach allows individualized target refinement and produces improved therapeutic outcomes. Genu, genus of the corpus callosum; Mid-SCC, mid subcallosal cingulate; Ac, anterior commissure; mF10, medial frontal Brodmann Area 10; ACC, anterior cingulate cortex; aTh, anterior thalamus; vSt, ventral striatum; Fr-st, frontal striatal fibers.

One proposed mechanism of DBS in reducing features of treatment resistant depression is modulation of a multi-region network converging at the SCC (Figure 4). Structural connectivity analysis of SCC DBS confirms the SCC as a critical node within this specified “network,” as small differences in stimulation location can generate substantial differences in activated fibers. Recent studies have further confirmed which of these pathways are necessary for clinically significant effects of DBS. These pathways can now be prospectively characterized in individual patients using DBS parameter models coupled to structural connectivity analyses (Riva-Posse et al., 2014; Choi et al., 2015).

Close clinical monitoring and systematic long-term follow-up using small experimental cohorts outside of industry-sponsored trials have further provided new perspectives on the time course, trajectory and sustainability of DBS-mediated effects (Crowell et al., 2015). Notably, patients often experience contact-specific changes in mood, attention, psychomotor speed, and autonomic reactivity with initial testing during electrode implantation surgery. Importantly, these acute behavioral effects appear predictive of long-term response. Recent implementation of real-time recording of SCC LFP during acute testing and ongoing therapeutic DBS using the prototype Activa PC+S DBS system is providing a first-in-human view of differential SCC LFP changes mediating immediate, sub-acute, and chronic DBS-induced antidepressant effects at the neural level. LFPs measured at the site of stimulation combined with concurrent high density EEG will further enable characterization of clinically relevant network-wide effects. Findings from this small exploratory study will potentially provide new metrics to further improve precision of surgical targeting as well as new algorithms for DBS delivery beyond current methods. Validation of relationships between local and network-wide changes with the differential time course of recovery in specific clinical features will lay the foundation for sensing signals for next generation neurostimulation systems.

**Highlights**

- Initial selection of the SCC as a putative DBS target was principally based on converging findings from resting-state PET imaging studies of conventional antidepressant interventions.
- Recent work now is focused on refinement and optimization using multimodal methods combined with real-time behavioral and physiological metrics, providing a more precise identification of the optimal target location in real time.
- Modulation of a multi-region network converging at the SCC is a proposed mechanism of action for DBS in reducing features of depression.
- Validation of relationships between local and network-wide changes with the differential time course of recovery in specific clinical features will lay the foundation for sensing signals for next generation neurostimulation systems.

### Clinical assessment and management of tremor

**Figure 5:**
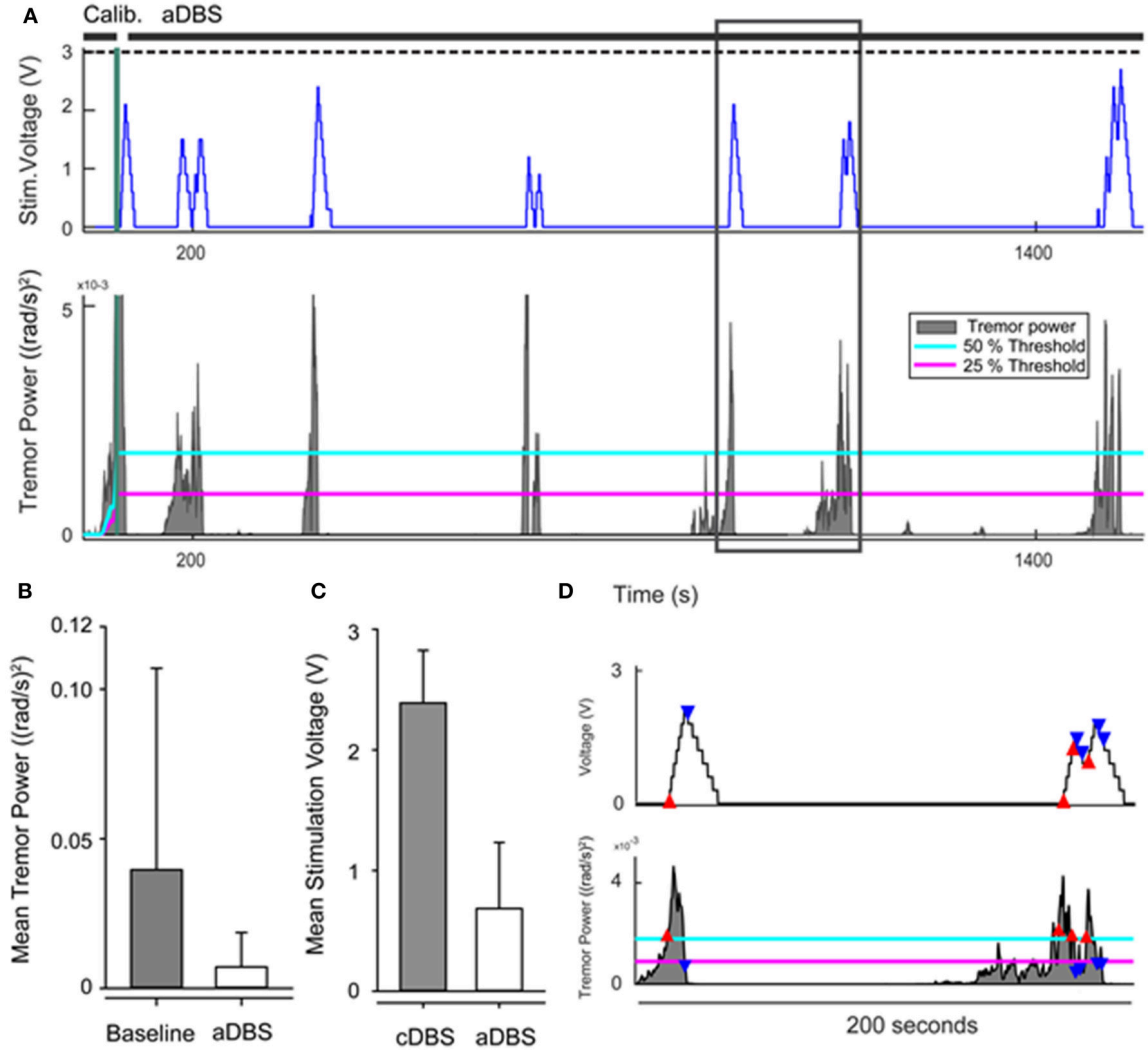
**(A)** Example of adaptive stimulation voltage (top row) and tremor power with 25% (magenta) and 50% (cyan) thresholds of the control policy algorithm (bottom row). Black horizontal lines above upper panel indicate timing of calibration and closed loop DBS (aDBS). Dashed black line shows level of clinical stimulation voltage. **(B)** Comparison of mean tremor power at baseline and during aDBS across the group. **(C)** Comparison of average stimulation voltage during open loop continuous (cDBS) and aDBS for the group. **(D)** Insert to **(A)** showing the timing of the aDBS decision tracking. When tremor power exceeded the upper threshold (red triangles), the stimulation voltage increased. When tremor power fell below the lower threshold (blue triangles), stimulation voltage decreased. Stimulation voltage remained unchanged if the tremor power level remained between lower and upper thresholds.

Tremor-dominant PD patients have been shown to have a functional correlation of their tremor and beta-band signals as measured by LFP. The resting state beta band may be attenuated during periods of tremor in PD. Resolution of tremor results in re-emergence of the beta band (Little and Brown, 2012). This suggests that beta band power may be viable as a kinematic control variable to drive closed loop DBS for tremor, as diminished beta band power during tremor could be assumed to signal a decrease in closed loop DBS. However, the activity-dependent fluctuations in the beta-band power limit its use a sole control for closed loop DBS in PD tremor. Bronte-Stewart and colleagues (Malekmohammadi et al., 2016) reported the efficacy of closed loop STN DBS to control resting tremor, using a kinematic measure of tremor power from use of a wearable Bluetooth enabled smart watch (LG G-watch; Figure 5).

In their study, baseline tremor recordings were performed, from which maximum tremor power was calculated. Closed loop DBS was driven by real-time measurement of tremor: when tremor intensity exceeded 50% of the maximum baseline tremor power, the control policy algorithm commanded an increase in DBS voltage at a predetermined safe ramp speed; when tremor intensity fell below 25% of the maximum tremor power, voltage was decreased. Using this model it was noted that the rate of change in stimulation voltage (if decreased quickly) could be correlated to occurrence of rebound tremor. Consequently the rate of decreasing voltage was set at 0.5 times the ramp (or increase) rate. Overall, the mean tremor power significantly decreased by 36.6% (*p* = 0.014) during closed loop DBS, and the mean voltage used was 76.4% lower than that used during continuous open loop DBS (*p* = 0.02). On average, closed loop DBS was “on” for only 51.5% of the time (*p* = 0.002), but there was a significant variation among subjects in the duration and average voltage required for effective stimulation.

This study provided proof of concept that real time kinematic measurement of tremor represents a safe, tolerable and efficacious method to drive STN DBS for tremor in PD. This strengthens prior findings of pilot trials using a neural control variable to drive closed loop DBS in the treatment of PD (Little et al., 2013; Rosa et al., 2015), and provides further support for the use of kinematic controls to supplement LFP input in developing personalized closed-loop DBS systems.

#### Development and use of algorithms in closed loop systems—a focus on tremor

In current clinical practice, DBS treatment involves open loop control. The stimulation parameters are pre-set for each patient, and do not automatically adjust to the presence or absence of symptoms, side effects or other patient-specific variables. The result is excessive battery consumption, as well as the possibility for undesirable side effects. Work by Chizeck and colleagues has produced a platform for investigating the control of DBS (Herron and Chizeck, 2014), which has now being employed by other groups. One mobile, wireless version consists of a set of worn inertial and electromyography sensors that communicate via Bluetooth to a host application running on a smartphone, smart watch or laptop. Using sensed data, the host application initiates control decisions, including enabling or disabling stimulation or modifying individual stimulation parameters (voltage, pulse width, frequency). These control signals are then transmitted by Bluetooth to a Medtronic Nexus™ system, which relays packets and control on a hardware and software modification of the clinician programming unit, driving a FDA approved and implanted DBS system (the Medtronic Activa PC+S)™(Herron et al., 2015; Houston et al., 2015; Malekmohammadi et al., 2016). An alternative, fully implanted system that is currently under development uses implanted cortical electrodes (connected to the DBS) to measure local field potentials (along with the deep brain electrode), as indicators of tremor and/or patient intentions and stimulation adjustment requests. These systems are being evaluated on patients with essential tremor and PD (Houston et al., 2015). This represents a practical implementation of a brain computer interface—BCI (i.e., which can be used for voluntary BCI-triggered stimulation adjustment by the patient; Thompson et al., 2016). These platforms also provide an opportunity for collection of tremor and stimulation data for extended periods of time, which will be vital toward gaining further insight to both the neurological basis of tremor, and issues related to the long term viability and use of these devices (Brown et al., 2016).

**Highlights**

- Activity-dependent fluctuations in the beta-band power in STN limit its use a sole control for closed loop DBS in PD tremor.
- Kinematic input can be processed by a laptop or smartphone that produces control signals that are then transmitted via Bluetooth to a Medtronic Nexus system, which relays packets and control on a hardware and software modification of the clinician programming unit, driving a FDA approved and implanted DBS system.
- Combination of kinematic input in a closed-loop DBS system resulted in tremor control, but there was considerable variation among patients.
- Future directions include the development of fully implanted closed loop DBS systems.

### Development of a closed loop system for tourette syndrome

(See Section Tourette Syndrome).

## DARPA systems based neurotechnology for emerging therapies (SUBNETS) research programs updates

### Introduction

The goal of the Defense Advanced Research Projects Agency (DARPA) Systems-Based Neurotechnology for Emerging Therapies (SUBNETS) project is to develop closed-loop DBS projects that will address the multiple neuropsychiatric problems occurring in the veteran and general population, including post-traumatic stress disorder (PTSD), traumatic brain injury, depression, anxiety, chronic pain, and substance abuse. In a recent article, Vigo and colleagues estimate from published data, that the global burden of mental illness is 32.4% of years lived with disability (Vigo et al., 2016).

Currently available treatments (e.g., pharmacological and psychological therapies) can be helpful for some patients. However, some patients are left with partial or no response to such intervention(s). Brain stimulation offers notable promise in treating these patients, as there are already FDA approved indications for the use DBS in treating other neuropsychiatric conditions such as OCD (see: http://www.accessdata.fda.gov/cdrh_docs/pdf5/H050003b.pdf). To address this clinical problem, DARPA currently supports East Coast and West Coast Research Teams that are engaged in key projects focusing upon one or more areas of state-of-the-art DBS techniques and technologies. Sections East Coast Research Team Updates and West Coast Research Team Updates summarize their unpublished work.

### East coast research team updates

Transdiagnostic Restoration of Affective Networks by System identification and Function Oriented Real-time Modeling in Deep Brain Stimulation (TRANSFORM DBS), the DARPA 5 year, funded program at Massachusetts General Hospital (MGH) is currently in its second year.

**Figure 6:**
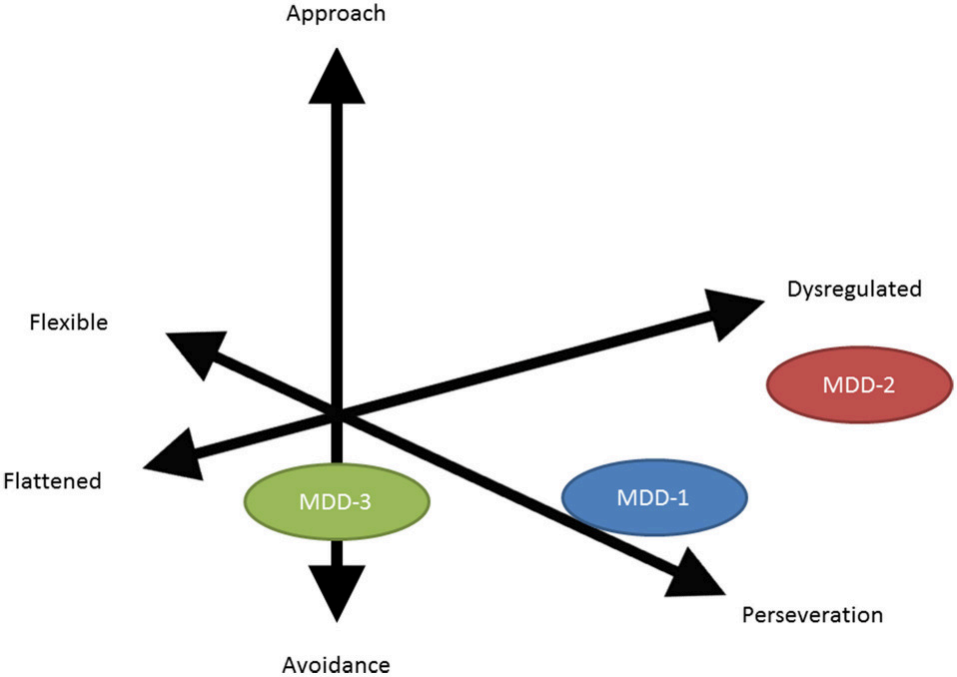
**Vector diagram illustrating the difference between a categorical diagnosis (in this instance MDD or major depressive disorder) and a symptom based or behavioral based domain assessment**. The limitation of the categorical diagnosis analysis is that it can average and thereby diffuse genuine subgroup (behavioral domain) therapeutic effects.

A recurring concern when attempting to categorize neuropsychiatric disorders is that most patients have co-morbid conditions that present with considerable variability. To decrease the effects of co-morbidity on analysis, the TRANSFORM DBS group employed a trans-diagnostic approach that focuses on behavioral domains rather than categorical diagnosis or co-morbidity assessments (Figure 6). Behavioral tests were developed to quantitate the severity of each domain, and these findings were used to guide treatment.

In collaboration with Draper Laboratory (Cambridge, MA), the group developed a modular, flexible implantable system that allows 320 simultaneous recordings. The identification of the deep electrode implantation site is determined by structural and functional assays relating to the different behavioral components. Studies thus far have been on non-human primates, with plans to extend investigations to humans in the epilepsy-monitoring beginning in summer 2016.

### West coast research team updates

Recent animal and human studies of brain connectivity have fostered a mesoscale network approach to interpreting and understanding mechanisms of neurocognitive function and dysfunction (Yuste, 2015).

This network-based construct suggests that psychiatric disorders may be related to changes in the function and/or structure of particular neural nodes and inter-nodal connectivity. Given the plasticity that has been demonstrated in neural circuity, a basic premise of SUBNETS is that DBS can be employed as a tool to facilitate re-modeling of brain architecture on micro to mesoscales.

The West Coast Team—at the UCSF—presented their initial work “mapping” the frontal and pre-frontal cortical areas by advancing an electrode grid under intra-operative fluoroscopy in PD patients undergoing DBS. At each cortical area, they stimulate and record the electrophysiological and clinical changes (mood states). The results show significant variability among patients, but indicate a possible correlation between the recorded signals and different mood states.

The next phase of this DARPA project will focus on chronic recording and stimulation in PD patients who have moderate psychiatric co-morbidities.

**Highlights**

- The goal of the DARPA's SUBNETS project is to develop closed-loop DBS projects for multiple high-burden neuropsychiatric disorders: PTSD, anxiety, depression, substance abuse, and pain.
- Two projects were discussed with different approaches in identifying DBS closed-loop systems:
  ◦ East Coast Group
    ■ Using behavioral domains (avoidance, perseveration, etc.) rather than categorical diagnoses (depression, anxiety, PTSD, etc.) as endpoints.
    ■ Using a custom-built modular and flexible implantable system allowing 320 simultaneous recordings.
    ■ Work so far has been on non-human primates with human studies planned to start in summer 2016.
  ◦ West Coast Group
    ■ Using intra-operative grid mapping of the frontal and pre-frontal cortical areas to assess their effect on affective states in patients with PD undergoing DBS.
    ■ Noted significant variability among the different patients.
    ■ Work so far has been on intra-operative recording, next phase will use chronic recordings.

## Development in technology and application

### Closed-loop DBS

**Figure 7:**
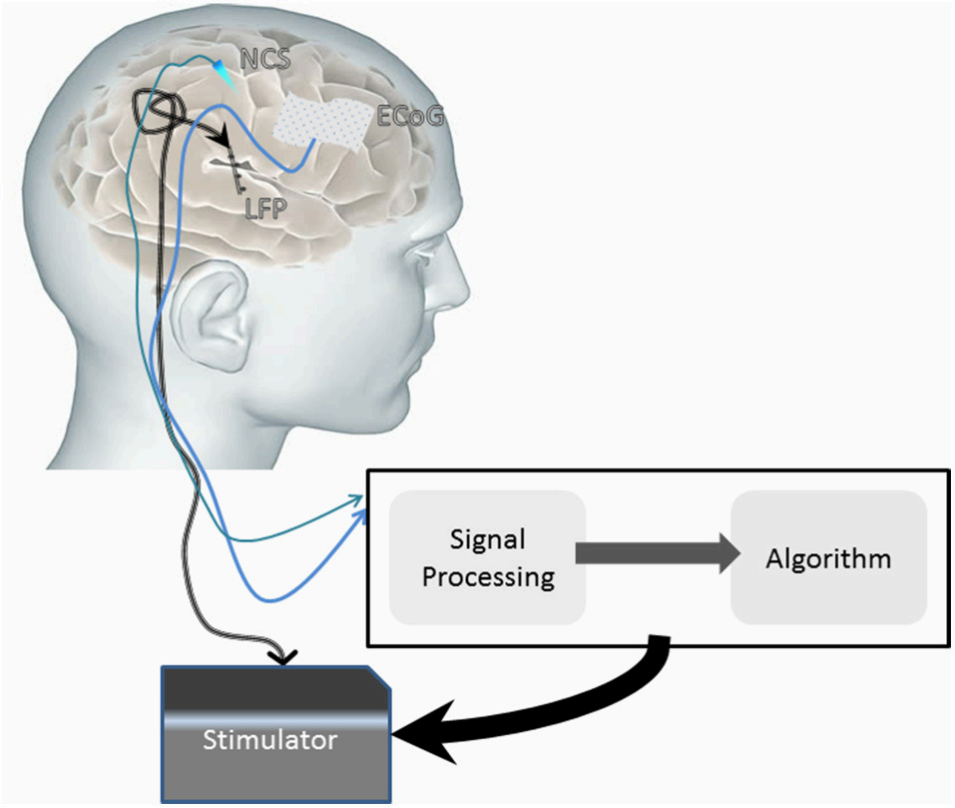
**Diagrammatic representation of a possible closed-loop DBS system comprised of sensors (e.g., ECoG, neurochemical sensors and local field potential sensing through the implanted electrodes) that influence the stimulator (actuator) signal**. The sensed signal is classified, and with use of an implementation algorithm, can influence the stimulator output to induce therapeutic effects.

Much of the hardware of DBS technology has been adapted from the cardiology field. A major limitation to ongoing refinement of DBS technology is a somewhat limited understanding of its mechanism(s) of action (Herrington et al., 2016). As noted in Section Introduction, open loop DBS does not respond to variations in the patient's state and disease progression but rather produces a pre-programmed output stimulation. This can result in a suboptimal outcome as optimization of the output stimulation has to be done by separate visits to provider clinics, usually many weeks apart. The closed-loop DBS system offers a solution by allowing integration of feedback signals to continuously modulate the output stimulation using an algorithm. The development of these advanced systems involves the construction of a number of components that are reliant, at least in part, upon feedback and feed-forward integration. The system comprises sensors that are connected to an actuator through a classifier and control policy (Figure 7).

Recent technological advances allow multi-modality sensing. Many of these modalities were discussed in prior sections and include LFP, ECoG, and neurochemical sensing modalities. There are multiple limitations to the current recording/sensing implantable technology. One, the signal sensed and recorded by implantable sensors has a lower quality than the one measured by stand-alone devices that are non-implantable and can be used only intra-operatively for a short period of time. Two, the signal measured is of relatively low amplitude. Three, identification of the appropriate signal to sense is still evolving and not clear for most indications (refer to Sections Tourette Syndrome, Development of DBS Sensors, Parkinson's Disease, Clinical Assessment and Management of Tremor). Four, there is a need to develop mechanisms to distinguish between the feedback and stimulation signals.

The feedback signal is transmitted from the sensors to a classifier system. The role of this system is signal processing, converting the raw signal into classified data that will be recognized by the algorithm. The latency of the signal transmission for analysis, though improving, is still a limiting factor in building closed loop DBS systems. The most complex component of developing algorithms for closed loop DBS is to modulate the output signal from the actuator (DBS stimulation) in order to affect the outcome toward the desired state. This is an area of considerable research. Section Clinical Assessment and Management of Tremor exemplifies the development of a classifier system based on the amplitude of the tremor and a relatively simple algorithm that modulates the output signal.

As with any biomedical technology, safety is an important aspect of closed loop DBS, and therefore a first objective is determining safety limits for the algorithms. A second and related consideration is the development of facile mechanisms to allow the patient and/or clinician to deactivate the closed-loop system, and/or engage a “default—safety mode” open loop system.

**Highlights**

- The DBS field is moving from the use of systems of continuous stimulation to more adaptive, closed-loop systems.
- To facilitate such progress, it will be important to address and resolve a number of issues, including:
  ◦ Improving recording and feedback signal acquisition.
  ◦ Improving latency time from signal sensing to analysis.
  ◦ Developing classifier systems that allow signal processing.
  ◦ Generating valid and safe algorithms of closed-loop function(s).
  ◦ Identifying markers of neural response.
  ◦ Identifying appropriate stimulation responses.
  ◦ Understanding and developing patient specific parameters for precision closed-loop DBS.

### Electrical current shaping

The most commonly used lead design in DBS systems includes four (4) ring-shaped contacts. In monopolar settings, each of these contacts produces a spherical electrical field. This can be problematic in cases when the lead is not optimally positioned in the target zone, as resultant stimulation induces side effects evoked by stimulation of off-target tissue(s) (Deuschl et al., 2006). To maintain clinical benefit while minimizing side effects, practitioners tend to modify the shape of the electrical field. Different approaches have been used, including bipolar stimulation, double monopolar stimulation, and/or interleaving settings.

Given the demonstrated importance of site and directional specificity of DBS electrical fields, new lead designs that allow current shaping/steering have been developed. The “directSTIM” lead, a design by Aleva Neurotherapeutics (Lausanne, Switzerland), divides each contact ring into 3 sub-compartments that can be individually stimulated (Hariz, 2014). Another, “SureStim,” developed by Sapiens (Eindhoven, the Netherlands), has 32 contacts distributed evenly (Contarino et al., 2014). A third, Vercise PC, produced by Boston Scientific and recently approved for use in Europe (September 2015), uses an 8-contact directional lead—the VANTAGE study (Timmermann et al., 2015). These designs allow current to be shaped away from unintended targets while maintaining a larger therapeutic window to the intended target site(s). Some limitations to these designs arise from the electrical properties of the system. For example, decreasing the surface area of the active contact will result in increased impedance and therefore increased power consumption. As well, impedance variation between different smaller contacts will passively dictate current distribution when more than one contact is simultaneously active if independent current sources are not employed.

To date, published data, as derived from use of the commercially available Vercise PC, has been limited to acute intraoperative settings, with only limited information available about the effects and efficacy of current steering in clinical care. However, unpublished data were presented at the Think Tank that illustrated the feasibility and improved therapeutic window of steered current, STN DBS in PD patients using the Vercise PC system in the clinic setting.

We posit that current steering/shaping approaches offer promise to improve the clinical outcomes of DBS, by allowing a wider therapeutic stimulation window, especially in those cases where lead placement may be difficult, and/or less than precise. For example, if a lead is targeting the STN in a PD patient but was noted post-operatively to be more lateral than initially planned, conventional stimulation will not only stimulate STN but also the adjacent internal capsule. This will produce a low threshold of stimulation to side effects thus providing only sub-optimal control of PD symptoms. By steering the current away from the internal capsule, a higher threshold of stimulation can be tolerated resulting in a better clinical outcome (Hariz, 2014).

**Highlights**

- A challenge facing the use of conventional DBS leads is delivery of the electrical current to the desired region while avoiding side effects by stimulating undesired areas.
- One approach proposed to decrease the undesired area stimulation is by using current steering
  ◦ Multiple lead designs are now being investigated.
- Unpublished results of prospective in-clinic current steering testing were presented that showed improved outcomes with STN current steering.

### Neurosurgical technique updates in DBS

The accuracy of surgical placement of the DBS electrode(s) at the specified anatomical targets is important. Although stereotactic techniques combining preoperative image-based planning with intraoperative recording and test stimulation are well-developed, these approaches carry risks including intracranial hemorrhage (1–3% both symptomatic and asymptomatic with <1% symptomatic), seizures (~1%), leak of cerebrospinal fluid (1–2%), and infection (2–3%) (Videnovic and Metman, 2008; Patel et al., 2015). While rates of adverse events appear to be quite low, in reality, these rates have been shown to increase to ~5% when data are prospectively and systematically collected (Burdick et al., 2010). Innovations in the surgical delivery of DBS include the use of intraoperative magnetic resonance imaging (Ostrem et al., 2013; Chabardes et al., 2015) to identify and guide electrode placement, and the use of frameless stereotaxy to enable more accurate surgical access and reduce burden and risks incurred by the operative hardware (Khan and Henderson, 2013). These are yet nascent, and the benefit and effect(s) of such innovations remains unknown.

Another surgical innovation in DBS lead implantation is the development of intravascular DBS electrodes. The feasibility of intravascular electrodes for both neural stimulation and recording and stimulation has been demonstrated. Electrodes positioned temporarily within intracranial vessels enabled recording of both spontaneous and evoked EEG-like electrical activity (Driller et al., 1969), and this work was recently extended to multi-electrode recordings using a chronically implanted stent-like device (Oxley et al., 2016). Stimulation through the blood vessel wall is also possible, an example being endovascular stimulation of the vagus nerve (Nabutovsky et al., 2007), and a recent simulation analysis demonstrated comparable patterns of model nerve fiber activation between an intravascular electrode and traditional stereotactically-positioned DBS leads (Teplitzky et al., 2014). This is not to suggest that intravascular methods are without risk; however, such innovations may provide a less-invasive approach to both record and stimulate deep in the brain and thus, represent new way to deliver DBS.

**Highlights**

- Significant advances have improved neurosurgical techniques of DBS implantation, but the complication rate remains ~5%.
- Intravascular stimulation may prove to be a viable, alternate method for delivering DBS.

## New clinical applications of DBS

### DBS for treatment of addiction

This year, a case was presented at the Think Tank of a female patient with severe OCD (displaying excessive cleaning behavior) who gained appreciable therapeutic benefit (i.e.,—reduction of compulsive behaviors and obsessive ideation) following nucleus accumbens DBS (Mantione et al., 2010). Of particular note however, was that this patient also was able to quit smoking and lost an appreciable amount of weight (she was obese) following DBS implantation. Although, it remained unclear whether these latter two effects were directly due to DBS or were artifacts, it was speculated that DBS may have effected change in neural circuitry mediating obsessive ideation and/or compulsive-type behaviors that subserve over-eating and smoking. The effect of the nucleus accumbens stimulation on addiction (cigarette smoking) in this OCD patient prompted interest in considering this target for treatment of addiction without co-morbid OCD. This has been reinforced by the identification of nodes and networks in the brain associated with addiction (Volkow et al., 2016).

This has prompted a funded trial of DBS targeting the nucleus accumbens to treat 8 heroin-addicted patients. Multiple cortical and deep brain recording sessions were performed while exposing the patients to either neutral or addiction-themed images. Change in signal intensity with cortico-basal coherence was used to identify the appropriate stimulation contact. Identifying the appropriate stimulation setting was achieved by asking the patient to engage in heroin “freebasing” behavior (using real heroin), and to rate his/her experience with each of the different settings used. At this writing, two patients have been treated, and decreased addiction behavior was noted in both patients with DBS stimulation. To be sure, these results are preliminary, and continued work in this study—and others—will be required to more accurately address and define the role and value of DBS in treating addition disorders.

**Highlights**

- The “optimal” target for DBS in addiction is not established; however, given extant data supporting the efficacy of nucleus accumbens-directed DBS in treating OCD, and similar cognitive and conative features of compulsive and addictive behaviors, the nucleus accumbens has been considered as a possible target.
- A study in the Netherlands that employed bilateral nucleus accumbens DBS implantation to treat heroin addiction demonstrated possible therapeutic benefits. At this point, results are preliminary, and continued work in will be required to more accurately address and define the role and value of DBS in treating addition disorders.

### Use of DBS to treat post-traumatic stress disorder

Dis-inhibition and propagation of fear responses appear to be cardinal neuro-cognitive features of PTSD (Furini et al., 2014). Extinction of fear responses involves engagement of the basolateral nucleus of the amygdala (BLn) and the medial prefrontal cortex (mPFC) (Marek et al., 2013). Fear responses (to even inert stimuli) can become heightened if this network is compromised. In this event, progressive psychotherapeutic approaches, such as progressive desensitization and/or stimulus immersion are less likely to succeed. Pharmacotherapy using benzodiazepines, while somewhat effective, is burdened by side effects (inclusive of sedation, tolerance, and withdrawal), and other pharmacological approaches (e.g., azapirones; beta-receptor antagonists) have been shown to be only nominally effective (Ravindran and Stein, 2010). In these cases, DBS of the amygdala may be useful to suppress abnormal activity within amygdalar-prefrontocortical circuits, and “re-set” the inhibitory tone necessary for fear extinction and reduction of PTSD symptoms.

Langevin presented results of a 1-year study of a patient with treatment-resistant PTSD who received BLn DBS. The patient exhibited and reported significant improvements in all domains of PTSD assessed (Langevin et al., 2016). In particular, the patient reported and evidenced improved quality and quantity of sleep (without nightmares), overall reduction in anxiety, fear and irritability, and improvement in interpersonal interactions with family members and work colleagues. Patient scores on the clinician administered PTSD scale decreased in excess of 40%. There have been no treatment-related adverse events and, in particular, monthly EEG studies have shown no evidence of seizure or epileptiform activity. In addition, the monthly EEG has shown progressively more sleep activity and improved sleep architecture, with the patient showing increased deep sleep that consistently occurs earlier in the patient's sleep cycle. Pre-operatively, the patient had undergone a fludeoxyglucose (FDG) PET imaging studies, both at rest and under symptomatic conditions during an exposure therapy session. These studies revealed increased metabolic activity in the amygdala during the symptomatic phase, as compared to the resting phase (Langevin et al., 2016). The FDG PET study was repeated 1 year after initiation of BLn DBS; post-treatment PET showed no difference in amygdalar metabolic activity between the resting and the symptomatic phase. This finding is consistent with patterns of amygdalar activity during fear extinction, suggesting the efficacy of BLn DBS in restoring a more normal pattern of activity in amygdalar networks involved in cognitive and behavioral aspects of fear that are representative of PTSD. The study is continuing, with ongoing recruitment toward a target enrollment of six patients.

**Highlights**

- A case was presented to support the possibility of using DBS targeting the amygdalar BLn to reduce signs and symptoms of PTSD
  ◦ Prior to DBS, the patient had disrupted sleep quality and quantity, night terrors, OCD-like symptoms, and manifest social disturbances. Following BLn DBS, the patient reports feeling calmer, evidences improvement in sleep architecture and quality (as demonstrated by EEG and described through self-report), and describes improvement in social interactions.
- FDG PET studies revealed post-DBS normalization of metabolic activity in the amygdala metabolism.

### Use of DBS to treat clinical obesity

Clinical, morbid obesity is a significant public health problem, both in the United States, and worldwide (Nangunoori et al., 2016). The current standard of treatment for morbid obesity that is not responsive to dietary and lifestyle modification, or pharmacotherapy is bariatric surgery. However, bariatric surgery although certainly of clinical value, also poses a number of risks, and is not uniformly successful (Ho et al., 2015). In seeking alternatives to gastro-intestinal surgery, DBS has been proposed as a viable approach to affect hypothalamic mechanisms of hunger and satiety that may be dysfunctional in morbidly obese patients (Nangunoori et al., 2016).

To explore this possibility, three (3) patients with a history of bariatric surgery were recruited; each with a current body mass index (BMI) greater than 40 Kg/m^2^. The DBS target was the bilateral lateral hypothalamus and post-operative images confirmed successful electrode implantation in all cases. The patients were followed for 2.5 years. The primary goal of this study was to assess safety. There were no serious adverse effects reported and no evidence of autonomic dysfunction. There was a subjective report of decreased urge to eat. Although, the study was not designed to assess efficacy, the resting metabolic rate was measured using a metabolic chamber. It was noted that contacts centered in the lateral hypothalamus were associated with an increase in the resting metabolic rate. This, however, did not correlate with a consistent weight loss (Whiting et al., 2013).

Long-term follow-up data were presented; one patient dropped off due to delayed bariatric surgery complications. During the long-term follow-up period, multiple DBS settings (9 settings/day) were used to determine the optimal stimulation parameters required to elicit the greatest increase in resting metabolic rate. In one patient, BMI decreased from 46 to 38, while the other patient did not show any weight loss or change in BMI. However, both patients showed an increase in their resting metabolic rate. The effects of any stimulation paradigm were short-term. This was attributed to the “hedonic component of food seeking and the motivational processes that drive eating” (Whiting et al., 2013).

Future directions for studying the potential viability and value of DBS to treat certain forms of clinical, morbid obesity are centered upon the identification of other neurological targets (e.g., the nucleus accumbens; see also Section DBS for Treatment of Addiction, above; either singularly, or in combination with stimulation of the lateral hypothalamus), and the potential utility of employing closed-loop systems.

**Highlights**

- DBS to treat clinical morbid obesity has targeted the lateral hypothalamus in an attempt to restore balance in hunger and satiety states.
- In a limited study (*n* = 3), obese patients who had previously undergone bariatric surgery and still maintained a BMI > 40 Kg/m^2^ underwent DBS surgery targeting the lateral hypothalamus.
- The procedure was noted to be safe in the 3 patients tested.
- Two years post-operatively, assessments indicate an increase in the resting metabolic rate though this did not translate to consistent weight loss.
- Future directions are focusing upon identification of other and/or additional neuroanatomical targets for DBS to treat clinical, morbid obesity.

## Conclusion

Herein we have summarized the presentations and discussion(s) of the Fourth Annual DBS Think Tank. Policy and regulatory issues and proposed optimizations were discussed, multiple advances in the field were addressed, including updates on the state of research, database and data registry, developments in closed-loop DBS, the most current and novel applications of DBS, and advances in electro-neurochemical sensing systems. In sum, the field and applications of DBS are expanding, and to some extent this expansion may represent a change in the status and trajectory of DBS research and use in clinical practice. To assess participants' perspectives and attitudes toward the current and near-term future development in the field, an anonymous 40 question poll was sent online at the conclusion of the Think Tank. The questionnaire assessed the respondent's perceived position of DBS applications in disease states, neurotechnological principles, and emerging applications on the hype cycle graph.

**Figure 8:**
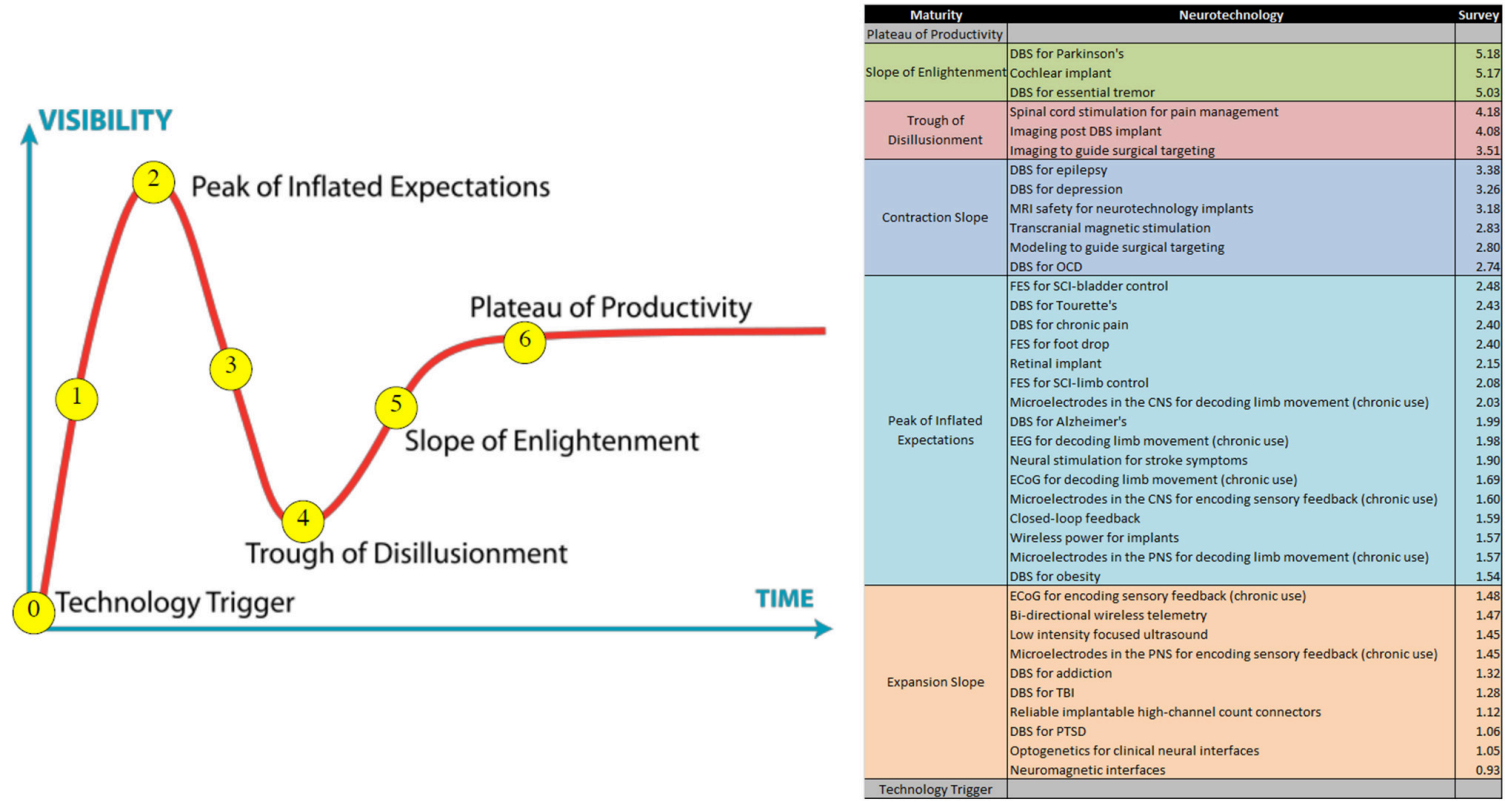
**Schematic results of an anonymous poll of ThinkTank participants to assess perceptions and attitudes about the current and near-term state of the DBS field**. On the left is a representation of the stages of technological development, known as the “hype cycle” graph. Participants in the Think Tank were asked to rank different DBS applications and other neurotechnologies on the “hype cycle.” Their responses were averaged and categorized, as depicted in the table on the right. Categories were assigned by rounding to the nearest whole number. Details in text. Figure adapted from: Jackie Fenn, “When to leap on the hype cycle,” Decision Framework DF-08-6751, Research Note, GartnerGroup RAS Services, June 30, 1999.

Thirty-six participants responded to the poll. These responses are depicted in Figure 8. Of note is in contrast to last year, participants' current perception of some uses/indications for DBS have slipped from the plateau of productivity to the slope of enlightenment (e.g., Parkinson's disease), others moved from the trough of disillusionment to the contraction phase (e.g., Depression), still others moved from the expansion slope to the peak of inflated expectations (e.g., Obesity).

In conclusion, the fourth Annual DBS Think Tank provided a nexus for the presentation of new developments and findings, discussion about the technology, research and practice of DBS, and speculation about—and proposals for—the future of the field. The future of DBS therapy will rely on continuing innovation and cooperation of key stake and shareholders, inclusive of scientists, engineers, physicians, ethicists, administrators and policy makers. The aim of the DBS Think Tank is to remain an important component of, and resource for contributions to this process and progress.

## Author contributions

AM, AG, AWS, BK, CB, CH, CV, DD, DDD, DR, FP, GG, GS, HW, HB, HM, HC, JL, JJ, JV, JO, JJS, KF, MR, MO, MP, PJR, PASi, PASt, TD, USA, WG fulfilled the authorship criteria by substantial contributions to the conception of the work, providing data for the work, revisiting it critically for important intellectual content, approving the final version, and agreeing to be accountable for all aspects of the work in ensuring that questions related to the accuracy or integrity of any part of the work are appropriately investigated and resolved.

WD, JG, PJR, JS, MSO fulfilled the authorship criteria by substantial contributions to the design of the work and the acquisition, analysis and interpretation of data for the work, drafting the work and revising it critically for important intellectual content, approving the final version to be published and agreeing to be accountable for all aspects of the work in ensuring that questions related to the accuracy or integrity of any part of the work are appropriately investigated and resolved.

## Funding

National Institutes of Health, National Parkinson Foundation, Michael J. Fox Foundation for Parkinson's Research and the Tourette Association of America provided funding for the previous studies presented in this paper.

### Conflict of interest statement

AM: Consulting fees and honoraria from Medtronic; AG: Research agreement with Medtronic; BK: Receive research support from Medtronic and Boston Scientific; CB: authored intellectual property related to deep brain stimulation and other neuromodulation therapies. CB: has served as a consultant for St Jude Medical, Functional Neuromodulation, and Boston Scientific. DDD: Research support from Medtronic, Inc. FP: Consulting Fees: Consultant for Medtronic. GG: Sole proprietor of Quanteon, LLC, which makes electrochemical recording instrumentation for measures of neurotransmitters and metabolic molecules in the brain of laboratory animals; GS: Grant support from Functional Neuromodulation as Director of the Imaging Core Laboratory for the ADVANCE trial; HW: Receives funding for research from Medtronic; HM: Consultant and licensor of intellectual property to St. Jude Medical, Inc.; JG: serves as an appointed member of the Neuroethics, Legal and Social Issues Advisory Panel of the Defense Advanced Research Projects Agency (DARPA) working on the Systems Based Neurotechnology for Emerging Therapies (SUBNETS) and Restoring Active Memory (RAM) projects. He does not receive any financial compensation in this role; his views expressed in this manuscript do not necessarily represent those of DARPA or the United States Department of Defense. JG: has previously received research grants from the Department of Defense; United States Air Force Office of Scientific Research; Department of the Navy, Bureau of Medicine and Surgery and Office of Naval Research; JV: Fulbright Foundation; and the Nour Foundation. He has received royalties from Cambridge University Press, Linton-Atlantic Press, and CRC Press (for books on neuroethics and brain science). JG currently serves as Editor-in-Chief of the BioMed Central Online journal Philosophy, Ethics, and Humanities in Medicine, and Associate Editor of the Cambridge Quarterly of Health Care Ethics. In the past 12 months, he has received funding from an unrestricted research grant from Thync Biotechnologies; however, this grant terminated in in early 2016 also receives funds from the National Center for Advancing Translational Sciences (NCATS, UL1TR001409), National Institutes of Health, through the Clinical and Translational Science Awards Program (CTSA), a trademark of DHHS, part of the Roadmap Initiative, “Re-Engineering the Clinical Research Enterprise”; JL: Inventor on a patent application for DBS in PTSD; JV: Grants and personal fees from Medtronic Inc., Grants and personal fees from Boston Scientific, Personal fees from St. Jude, outside the submitted work; JO: Research Grant Support from Boston Scientific, St. Jude Medical; JJS: Consulting activities for Medtronic and St Jude Medical; KF: Research and fellowship support from Medtronic. Research support from St. Jude, Boston Scientific, NeuroPace, and Functional Neuromodulation. No personal remuneration from industry sources. MO: Funding in part from Medtronic; MP: Consulting fees from Medtronic, Inc., and St. Jude Medical; MSO: serves as a consultant for the National Parkinson Foundation, and has received research grants from NIH, NPF, the Michael J. Fox Foundation, the Parkinson Alliance, Smallwood Foundation, the Bachmann-Strauss Foundation, the Tourette Syndrome Association, and the UF Foundation. MSO: DBS research is supported by: R01 NR014852. MSO has previously received honoraria, but in the past >60 months has received no support from industry. MSO has received royalties for publications with Demos, Manson, Amazon, Smashwords, Books4Patients, and Cambridge (movement disorders books). MSO is an associate editor for New England Journal of Medicine Journal Watch Neurology. MSO has participated in CME and educational activities on movement disorders (in the last 36) months sponsored by PeerView, Prime, QuantiaMD, WebMD, MedNet, Henry Stewart, and by Vanderbilt University. The institution and not MSO receives grants from Medtronic, Abbvie, Allergan, and ANS/St. Jude, and the PI has no financial interest in these grants. MSO has participated as a site PI and/or co-I for several NIH, foundation, and industry sponsored trials over the years but has not received honoraria. PAS: Consulting work for Medtronic Inc., and Boston Scientific; US patent related to closed loop deep brain stimulation in movement disorders. NIH (R01 NR014852 - PIs Butson and Okun; R01 NS096008 - PI Okun). TD: Medtronic employee, Medtronic Shareholder, Intellectual property in these areas. WG: Inventor on licensed patents on temporal patterns of deep brain stimulation and owns equity in Deep Brain Innovations, LLC. WD, PJR, UA, PS, PJR, MR, JS, HC, HB, DR, DD, JJ, and CH: declare that the research was conducted in the absence of any commercial or financial relationships that could be construed as a potential conflict of interest.

## Abbreviations

3-D: Three dimensional
AD: Alzheimer's disease
ADD: Attention deficit disorder
BCI: Brain computer interface
BLn: Basolateral nucleus of the amygdala
CM: Centromedian thalamus
CMS: Centers for Medicare and Medicaid services
CUA: Cost Utility Analysis
DARPA-SUBNETS: Defense Advanced Research Projects Agency - Systems-based Neurotechnology for Emerging Therapies
DBS: Deep brain stimulation
DTI: Diffusion tensor imaging
ECoG: Electrocorticogram
EQ-5D: European quality of life 5 dimensions
FDA: Food and Drug Administration
FDG: Fludeoxyglucose
GDP: Gross Domestic Product
GTS-QoL: Gilles de la Tourette Syndrome quality of life
HDE: Humanitarian device exemption
HFS: High frequency stimulation
HR-QoL: Health related quality of life
ICER: Incremental cost effectiveness ratios
IDE: Investigational device exemption
IIR: Investigator initiated research
IRB: Institutional review board
LFP: Local field potential
LFS: Low frequency stimulation
MCID: Minimal clinically important difference
mPFC: Medial Prefrontal cortex
NINA: Neurological information non-discrimination act
NNTI: National neurotechnology initiative
OCD: Obsessive-Compulsive disorder
PET: Positron emission tomography
PTSD: Post-traumatic stress disorder
QALY: Quality adjusted life year
QoL: Quality of Life
RNS: Responsive neurostimulator
ROR: Right of Reference
SCC: Subcallosal cingulate
SF-36: Short-form 36-item
STN: Subthalamic nucleus
TAA: Tourette Association of America
TRANSFORM DBS: Transdiagnostic Restoration of Affective Networks by System identification and Function Oriented Real-time Modeling in Deep Brain Stimulation
TRD: Treatment resistant depression
TS: Tourette Syndrome
TSA: Tourette Syndrome Association
UCSF: University of California at San Francisco
VNS: Vagal nerve stimulator
YGTSS: Yale Global Tic Severity Scale.
